# Fighting Fire with Fire: Immunogenicity of Viral Vectored Vaccines against COVID-19

**DOI:** 10.3390/v14020380

**Published:** 2022-02-12

**Authors:** Aiquan Chang, Jingyou Yu

**Affiliations:** Beth Israel Deaconess Medical Center, Center for Virology and Vaccine Research, Harvard Medical School, Boston, MA 02115, USA; achang7@bidmc.harvard.edu

**Keywords:** SARS-CoV-2, COVID-19, viral vectored vaccine, antibody, T cells

## Abstract

The persistent expansion of the coronavirus disease 2019 (COVID-19) global pandemic caused by severe acute respiratory syndrome coronavirus 2 (SARS-CoV-2) requires the rapid development of safe and effective countermeasures to reduce transmission, morbidity, and mortality. Several highly efficacious vaccines are actively being deployed around the globe to expedite mass vaccination and control of COVID-19. Notably, viral vectored vaccines (VVVs) are among the first to be approved for global distribution and use. In this review, we examine the humoral, cellular, and innate immune responses elicited by viral vectors, and the immune correlates of protection against COVID-19 in preclinical and clinical studies. We also discuss the durability and breadth of immune response induced by VVVs and boosters. Finally, we present challenges associated with VVVs and offer solutions for overcoming certain limitations of current vaccine regimens. Collectively, this review provides the rationale for expanding the portfolio of VVVs against SARS-CoV-2.

## 1. Introduction

The coronavirus disease 2019 (COVID-19) pandemic represents one of the most devastating global health challenges in human history. As of January 2022, SARS-CoV-2 had caused more than 350 million infections and 5.6 million deaths, straining health care systems and undermining social and economic domains [1]. At an unprecedented pace, academic laboratories, biotechnology companies, and regulatory agencies have developed and deployed several effective vaccines that have significantly curtailed morbidity and mortality in the global landscape. Vaccines based on viral vectors, which are genetically modified wild-type viruses, have been at the forefront of these collective efforts. These vaccines have demonstrated adequate safety profiles, strong immunogenicity characteristics, and most importantly, high levels of protection from severe COVID-19 disease.

**Figure 1:**
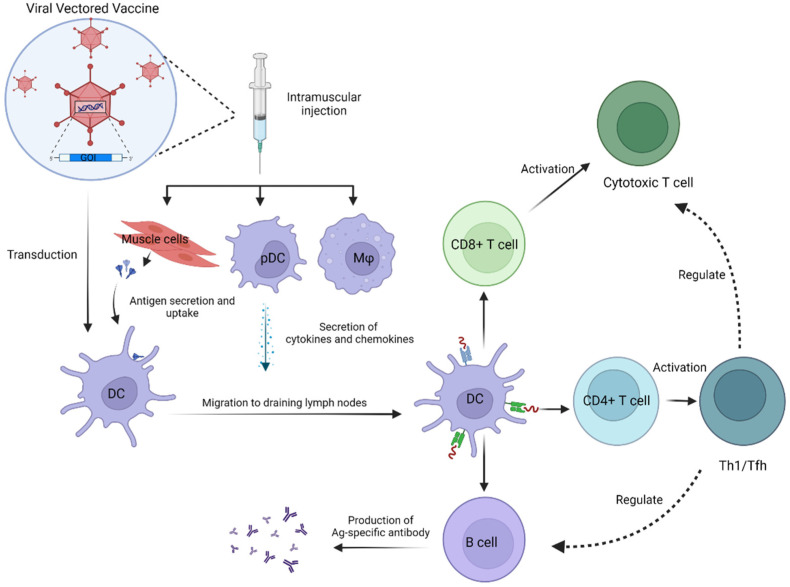
Viral vectored vaccines (VVVs) elicit innate and adaptive immune responses. Dendritic cells (DCs), macrophages, and plasmacytoid dendritic cells (pDCs) are sentinel cells that reside in tissues. Following intramuscular administration, viral particles transduce muscle cells and sentinel cells, leading to high levels of protein production (i.e., spike protein of SARS-CoV-2). DCs are present in various tissues and can capture, phagocytose, and digest antigen. As DCs upregulate pro-inflammatory cytokines, costimulatory molecules, chemokine receptors, and antigen presentation, they can traffic to the draining lymph node and prime naïve T cells by presenting them with specific antigen and costimulatory ligand. The activated T cells can proliferate and differentiate into effector or memory cells. A CD4+ T cell could differentiate into a helper T cell, such as a Th1 cell, but could also differentiate into a T follicular helper cell that assists B cells to produce antigen-specific antibodies. A CD8+ T cell could differentiate into a cytotoxic T cell that eradicates virally infected cells. While some memory T cells will persist, most effector T cells die over time. Abbreviations: GOI: gene of interest; DC: dendritic cell; pDC: plasmacytoid dendritic cell; Mφ: macrophage; Ag: antigen.

Viruses are intrinsically very efficient at entering target cells, delivering genetic instructions to key intracellular compartments, and driving protein expression [2]. Generally, the development of viral vectored vaccines (VVVs) entails the insertion of genes that encode heterologous antigens (e.g., spike proteins of coronaviruses) with or without modifications (Figure 1). The recombinant viral expression vector can then be evaluated in animal models to assess immune responses and protective effects against challenges with etiologic agents. Viral vectors display a number of key immunological features which make them promising vaccine platforms. They can efficiently transduce genes of interest into specific target cells and can elicit exceptionally potent antigen-specific antibody and, most importantly, T-cell responses without the need for an adjuvant (Figure 1) [3]. To date, numerous viral vectors have been exploited to combat widespread infectious diseases caused by clinically important viruses, including Ebola, HIV, influenza, Zika, and now SARS-CoV-2 [4]. Twenty-one percent of the 334 COVID-19 vaccines currently under preclinical and clinical development constitute viral vectors [5]. Most aim to mount a protective immune response against the SARS-CoV-2 spike (S) protein that mediates viral entry into host cells. Here, we present some prominent examples, with a summary provided in Table 1.

This review aims to illuminate the unique immunological features of viral vectors that have contributed to their burgeoning eminence as a vaccine platform. Specifically, we discuss adaptive and innate immunity afforded by VVVs and their correlations with disease protection from animal models and human clinical trials. We also discuss the challenges that VVVs pose for achieving optimal immunogenicity and strategies that have been pursued to overcome them. We will not address the virology of VVVs, which has been covered in detail in other reviews [6,7].

## 2. Immunogenicity

### 2.1. Humoral Immunity

Humoral immunity to SARS-CoV-2 is achieved through vaccination or natural infection. Neutralizing antibodies, which are a key component of humoral immunity, stand as one of the most important predictors of immune protection from SARS-CoV-2 infection. Viral vectors differ in their entry kinetics, replication capacity, and protein expression profiles, and thus the level of immune response and protection provided by VVVs can dramatically vary.

#### 2.1.1. Adenoviral Vector

Adenoviruses (Ads) are double-stranded, non-enveloped DNA viruses with more than 300 serologically distinct serotypes [8]. By using their viral receptors, Ads can target a broad range of host tissues for cellular infection, a key attribute that has opened up important avenues to design novel vaccine concepts and approaches against SARS-CoV-2 and other etiologic microbes [9].

In collaboration with the healthcare company Johnson and Johnson (J & J), researchers at Beth Israel Deaconess Medical Center developed a replication-defective adenovirus serotype 26 (Ad26)-based COVID-19 vaccine (Ad26.COV2.S, immunogen: S.PP), and investigated its immunogenicity in animal models [10,11]. In line with other Ad26-vectored vaccines [12,13,14,15], Ad26.COV2.S induced strong humoral and cellular immune responses. In golden Syrian hamsters, a single immunization of high dose (10^10^ viral particles (vp)) or low dose (10^9^ vp) of Ad26.COV2.S via the intramuscular route (IM) led to strong receptor binding domain (RBD)-specific binding antibody and neutralizing antibody (NAb) responses, and protected against SARS-CoV-2-induced weight loss, pneumonia, and mortality [10]. As early as 2 weeks after immunization, all vaccinated hamsters seroconverted and showed a substantial increase (by approximately 20-fold) in median NAb titers compared with sham control hamsters [10]. Additionally, a dose-escalation study in hamsters demonstrated that binding and neutralizing antibodies were induced in a dose-dependent fashion, as immunizations with 10^9^ or 10^10^ vp resulted in a 6-fold or a 10-fold increase in NAb titers compared to control hamsters, respectively [16,17]. In rhesus macaques, a single IM immunization of 1 × 10^11^ vp induced pseudo-virus and live virus NAb titers that were 20-fold greater than those of sham macaques at week 4 post-vaccination [11]. Of note, Ad26.COV2.S-vaccinated macaques exhibited immensely higher (>4 fold) median NAb titers than those found in the sera of convalescent humans and macaques [18]. A dose-titration study in macaques further revealed that a single low dose (2 × 10^9^ vp) was able to elicit binding and neutralizing antibodies 6 weeks post vaccination, contrasting with the higher dose regimens that produced antibodies as early as week 2 [19].

A phase 1/2a trial conducted in Belgium and the USA showed a single immunization of low dose (5 × 10^10^ vp) or high dose (1 × 10^11^ vp) of Ad26.COV2.S elicited increasing levels of antibody response during the 71 days of follow-up after immunization in the majority of vaccinees [20]. NAb titers against the ancestral strain WA2020 were detected in 90% or more of participants by day 29 after the first immunization, irrespective of dose or age, and reached 96% by day 57 with a further titer increase in young adults. Overall, a single immunization of Ad26.COV2.S elicited NAb titers that were similar to those found in convalescent sera by day 57 post-vaccination, and a homologous booster two months later further augmented titers by 2–3-fold [20]. In a smaller cohort involving 25 participants, the kinetics and magnitude of antibody responses were determined [21]. Binding and neutralizing antibodies were observed as early as 8 days after immunization, peaked on day 29, and maintained steady at least until day 71. Altogether, the Ad26.COV2.S vaccine is highly immunogenic in the young and the elderly. While lower doses may give a slower response, they have shown the capacity to generate antibody responses of sufficient magnitude. Hence, low-dose vaccination strategies may be favorable to ensure safety without sacrificing immunological potency.

Replication-defective adenovirus serotype 5 (Ad5) is another adenoviral-vectored vaccine that has been leveraged in response to COVID-19. In BALB/c mice, a single mucosal immunization of Ad5 encoding the full-length S protein (Ad5-nCoV) induced robust binding and neutralizing antibody responses and protected the lungs of mice from SARS-CoV-2 infection [22]. In C57BL/6 mice, a low-dose prime (LD, 1 × 10^6^ vp) with a standard dose boost (SD, 1 × 10^9^ vp) of an Ad5-based vaccine encoding S led to a 72-fold increase in NAbs and superior CD8+ T cell responses compared with the SD/SD regimen [23]. Indeed, this observation aligns with a recent ChAdOx1 nCoV-19 (AZD1222) clinical trial, in which a half-dose prime conferred greater protective efficacy than a standard-dose prime [24]. In addition, Ad26.COV2.S elicited similar levels of antibody responses, irrespective of high- (1 × 10^11^ vp) or low-dose (5 × 10^10^ vp) vaccination [20]. Therefore, there may exist a threshold for the induction of optimal protection, above which vaccinating with higher doses leads to immune exhaustion, and below which vaccinating with lower doses fails to generate sufficient immunity against pathogens. Further, Sanchez et al. mechanistically found that lowering the priming dose generated higher central memory CD8+ T cells and fewer exhausted T cells, which underpin dose-sparing as a strategy to improve recall response and vaccine accessibility [23]. In CD-1 mice, a prime-boost immunization of a dual antigen vaccine expressing modified SARS-CoV-2 S and nucleocapsid (N) proteins (hAd5 S-Fusion + N-ETSD) by the subcutaneous route (SC) elicited stronger anti-S IgG2a, IgG2b, and NAb responses compared with hAd5 S-WT; the researchers ascribed the improvement in antibody response to enhanced cell surface expression of S-Fusion [25]. In rhesus macaques, a 1 × 10^11^ vp SC prime immunization with two 1 × 10^10^ IU oral boosters elicited potent anti-S NAbs and protected animals from high titer SARS-CoV-2 infection [26]. In human adults, a single shot of Ad5-nCoV induced binding and neutralizing antibodies that peaked on day 28 post-vaccination [27,28]. Among the vaccinees, the seroconversion rates of binding and neutralizing antibodies were 90% and 50%, respectively (seroconversion was defined as a 4-fold increase in post-vaccination titer compared with baseline). In children and adolescents aged 6–17 years, a single injection of Ad5-nCoV was able to induce strong antibody responses without adverse reactions [29]. At present, Pfizer-BioNTech’s BNT162b2 mRNA vaccine is the only COVID-19 vaccine authorized for emergency use in children 5 through 11 years of age. Therefore, additional safe and effective vaccines are urgently needed to protect vulnerable populations (i.e., children, elderly, pregnant women, immunocompromised patients) and communities where the virus continues to circulate.

Since SARS-CoV-2 transmits efficiently via respiratory droplets and tends to gain entry at the respiratory mucosa, some speculate that administration through the respiratory tract (i.e., intranasal vaccination) is more likely to prevent infection at the portal of viral entry. For hAd5 S-Fusion + N-ETSD, IN prime + IN boost induces stronger humoral immunity than SC prime + IN or SC boost [25]. In BALB/c mice, a single SC or IN immunization of 1.5 × 10^10^ vp of Ad5.S1 elicited comparable, potent IgG and NAb responses [30]. In BALB/c mice, a single IN or IM immunization of 5 × 10^9^ vp of a rAd5 vaccine carrying a codon-optimized gene encoding S (Ad5-S-nb2) induced similar titers of serum IgG by day 28, and IN led to stronger IgA responses than IM [31]. A phase 1 trial conducted in China showed that two doses of aerosolized Ad5-nCoV induced NAb responses in the majority of vaccinees [32]. Out of all regimens, a prime IM immunization of 5 × 10^10^ vp with an aerosolized booster of 2 × 10^10^ vp induced the strongest NAb and RBD-binding IgG responses; IM immunization also turned out to induce stronger IgA responses than aerosolized immunization. On the whole, these data suggest that immunization routes can affect antibody production, and aerosolized vaccines serve as an appealing alternative to IM immunizations for the induction of potent humoral immunity.

The Sputnik V (Gam-COVID-Vac) COVID-19 vaccine, which was developed in Russia, uses rAd26 and rAd5 vectors in a heterologous prime-boost format [33]. In addition to exploiting the robust immunogenicity of both Ad vectors, this approach strives to circumvent pre-existing Ad immunity which may impede the induction of potent humoral immunity. A phase 1/2 trial conducted in Russia evaluated the immunogenicity of “Sputnik light”, a single-dose vaccine based on rAd26 [34]. Unlike Ad26.COV2.S, which encodes prefusion-stabilized S, rAd26 carries the gene for native SARS-CoV-2′s S protein (rAd26-S). For seropositive individuals, a single 1 × 10^11^ vp immunization of rAd26 elicited higher NAbs than those from convalescent plasma; for seronegative individuals, rAd26 elicited lower NAbs than those from convalescent plasma [34]. Additional studies showed that a single 1 × 10^11^ vp immunization of rAd26 or rAd5 elicited NAb titers that were 2–4 fold lower than those from convalescent plasma, and immunizations of the heterologous vaccine based on rAd26 and rAd5 induced higher NAb titers compared with those in convalescent plasma [35]. Similarly, a homologous boost immunization of Ad26.COV2.S further amplified antibody titers, in which the two doses were administered two months apart [20]. Together, it is imperative to refine heterologous prime-boost regimens to potentiate humoral response and improve the immunogenicity of Ad-vectored vaccines.

Besides human Ad26 and Ad5 vectors, AstraZeneca/Oxford developed a chimpanzee adenoviral-vectored vaccine, ChAdOx1 nCoV-19 (AZD1222), which encodes the SARS-CoV-2 S protein [36]. In mice and pigs, a single immunization of 10^8^ IU of ChAdOx1 nCoV-19 elicited binding and neutralizing antibody responses; in particular, a homologous boost increased NAbs by about 1-log in pigs [37]. In rhesus macaques, a prime-boost IM immunization of 2.5 × 10^10^ vp of ChAdOx1 nCoV-19 led to 1-log higher IgG and NAb titers compared to a prime-only regimen, and protected animals from SARS-CoV-2-induced pneumonia [38]. In ferrets, a prime-boost regimen of 2.5 × 10^10^ vp of ChAdOx1 nCoV-19 elicited 1-log higher NAb titers than a prime-only regimen, and IM yielded higher NAb titers than IN [39]. In a phase 1/2 trial conducted in the UK, a single immunization of 5 × 10^10^ vp of ChAdOx1 nCoV-19 induced antibody responses that peaked on week 4 and persisted at least until week 8 [36,40]. A boost immunization further enhanced NAbs by 4-fold, which was similar to the level of those obtained in convalescent plasma [36].

In addition to the adenoviral-vectored vaccines that have advanced into the clinic, the use of simian adenoviruses as a platform for vaccine development has gained steady momentum. In mice and hamsters, a single dose of a chimpanzee Ad-vectored vaccine (ChAd-SARS-CoV-2) induced a robust and durable S-specific antibody response capable of neutralizing SARS-CoV-2 [41,42,43]. IN delivery induced approximately 6-fold higher antibody titers than IM delivery, in contrast to the ChAdOx1 nCoV-19 vaccine that elicited higher antibodies through the IM route in ferrets [41,42,43]. In BALB/c mice, a prime-boost IM administration of a novel Ad-vectored vaccine (Sad23L-nCoV-S) and its self-biomineralized form (Sad23L-nCoV-S-CaP) elicited potent humoral responses [44]. In cynomolgus macaques, a single IM immunization of 5 × 10^10^ vp of a gorilla adenoviral-vectored vaccine (GRAd-COV2) encoding a prefusion-stabilized S induced potent IgG and NAb responses [45]. Importantly, NAbs 10 weeks post-vaccination were higher than those found in convalescent sera of recovered COVID-19 patients [45]. A prime immunization of 5 × 10^9^ PFU of Sad23L-nCoV-S together with a boost immunization of 5 × 10^9^ PFU of a human Ad49-based vaccine encoding the full-length gene of S (Ad49L-nCoV-S) induced potent and durable NAb, anti-RBD, and anti-S responses in rhesus macaques [46].

#### 2.1.2. Alternative Viral Vectors

A broad spectrum of alternative viral vectors has also undergone extensive preclinical evaluation, including in vesicular stomatitis virus (VSV)-based vaccines. In hamsters, IM immunization of 2 × 10^7^ PFU of ConVac, a VSV-based vaccine expressing the S1 subunit of S, induced NAb titers that were 100-fold higher than those of sham control animals, and protected animals from SARS-CoV-2 replication in the lung [47]. Of note, similar levels of SARS-CoV-2-specific antibodies were observed in ConVac-vaccinated hamsters and a recovered COVID-19 patient [47]. Another study of a recombinant VSV-G-spike SARS-CoV-2 vaccine assessed how dosage (ranging from 10^4^ to 10^8^ pfu) affected antibody production [48]. While 10^8^ pfu possessed the strongest immunogenicity, a single-dose IM injection of 10^6^ pfu was able to induce robust NAbs and protect against high-dose SARS-CoV-2 challenge in hamsters [48]. A methyltransferase-defective rVSV-based vaccine expressing the full-length S induced higher SARS-CoV-2-specific NAbs in serum of hamsters than in plasma of convalescent COVID-19 patients [49]. In BALB/c mice, a single immunization of 10^6^ PFU of a replication-competent VSV-vectored vaccine expressing a modified form of the spike gene (VSV-eGFP-SARS-CoV-2) induced 3-fold higher NAb titers compared to sham control animals; a homologous boost resulted in a greater-than-88-fold increase in neutralizing activity [50]. Live attenuated measles virus-based vaccines expressing prefusion-stabilized S induced higher NAb titers in cotton rats, mice, and hamsters than those found in sera from convalescent COVID-19 patients [51,52]. Despite the strong immunogenicity observed in animal models, a phase I study that examined “V590”, an rVSV-vectored COVID-19 vaccine candidate developed by Merck, terminated due to low immunogenicity [53]. Likewise, the development of a measles virus-based COVID-19 vaccine by Merck named “V591” was discontinued due to a lack of immunogenicity [54].

A live-attenuated yellow fever 17D vaccine encoding a non-cleavable prefusion form of SARS-CoV-2 S (YF-S0) induced robust NAb responses in macaques, hamsters, and mice [55]; YF-S0 was also shown to protect hamsters from SARS-CoV-2 challenge. A single IN immunization of CVXGA1, a parainfluenza virus type 5-based vaccine that expresses S, elicited NAbs and RBD-binding antibodies in a dose-dependent fashion in mice [56]. In hamsters, a prime-boost immunization of CORAVAX, an inactivated rabies-vectored vaccine encoding S1, together with a TLR4 agonist, generated potent NAb responses and protected animals from SARS-CoV-2 challenge [57]. In BALB/c mice, a prime-boost regimen in a 3-week interval of a Newcastle disease viral-vectored vaccine that expresses wild type S or membrane-anchored S without the polybasic cleavage site elicited robust NAb responses [58].

Further, DNA viruses such as oncolytic herpes simplex virus-1 (HSV-1) and modified vaccinia virus Ankara (MVA) were utilized to develop COVID-19 vaccines. Intriguingly, the HSV-1-based vaccine, termed OV-spike, is not only immunogenic but was also shown to prevent SARS-CoV-2 infection in mice and inhibit tumor growth in tumor-bearing mice [59]. In BALB/c mice, one or two immunizations of an MVA-vectored vaccine expressing modified versions of S elicited potent antibody responses and protected animals from intranasal SARS-CoV-2 challenge [60]. Another MVA-based vaccine, MVA-SARS-2-S, was evaluated in BALB/c mice and was shown to inhibit SARS-CoV-2 replication, irrespective of whether high-dose or low-dose immunization was employed [61].

Collectively, these findings and observations suggest that immunogen design, modality, dosing, route of immunization, homologous or heterologous prime-boost approaches, and individual variations among vaccinees can all affect vaccine-mediated humoral immunogenicity.

**Table 1:** Clinical and preclinical studies of viral vectored vaccines against COVID-19.

Vaccine	Model	Regimen	Route of Administration	Humoral Immune Response	Cellular Immune Response	Reference
Ad26.COV2.S	Hamster	Single dose of 1 × 10^10^ vp	IM	Median ELISA titer: 4470 (week 4) Median NAb titer: 359 (week 4) RBD-binding Ab and neutralizing Ab titer > sham	N/A	[10]
	Mouse	Single dose of 1 × 10^10^ vp	IM	Binding Ab and neutralizing Ab titer: S.PP > S > sham	Th1-biased response	[17]
	Rhesus macaques	Single dose of 1 × 10^11^ vp	IM	Median NAb titer: 408 (week 4) Median NAb titers 4-fold higher than convalescent titers of macaques and humans	Th1-biased response	[11]
	Human	High dose: 1 × 10^11^ vp Low dose: 5 × 10^10^ vp Cohort 1a: 18–55 years of age Group 1: placebo/placebo Group 2: low dose/placebo Group 3: low dose/low dose Group 4: high dose/placebo Group 5: high dose/high dose Cohort 3: ≥65 years of age Group 1: placebo Group 2: low dose Group 3: high dose	IM	Binding Ab (ELISA units/ml) (GMC): Cohort 1a Baseline: all below LOQ (<53) Day 29: <53 (Group 1) 478 (Group 2) 586 (Group 3) 625 (Group 4) 788 (Group 5) Day 57: <53 (Group 1) 660 (Group 2) 754 (Group 3) 873 (Group 4) 1100 (Group 5) Day 71: <53 (Group 1) 600 (Group 2) 1677 (Group 3) 951 (Group 4) 2292 (Group 5) Cohort 3 Baseline: all below LOQ (<53) Day 15: <53 (Group 1) 122 (Group 2) 141 (Group 3) Day 29: <53 (Group 1) 312 (Group 2) 350 (Group 3) Neutralizing Ab (IC50) (GMT): Cohort 1a Baseline: all below LOQ (<58) Day 29: <58 (Group 1) 224 (Group 2) 224 (Group 3) 215 (Group 4) 354 (Group 5) Day 57: <58 (Group 1) 310 (Group 2) 288 (Group 3) 370 (Group 4) 488 (Group 5) Day 71: <58 (Group 1) 321 (Group 2) 827 (Group 3) 388 (Group 4) 1266 (Group 5) Cohort 3 Baseline: all below LOQ (<58) Day 15: <58 (Group 1) 212 (Group 2) 172 (Group 3) Day 29: <58 (Group 1) 277 (Group 2) 212 (Group 3)	CD4+ Th1 cells Cohort 1a Day 15: 0% (Placebo) 76% (low dose) 83% (high dose) Cohort 3 Day 15: 0% (Placebo) 60% (low dose) 67% (high dose) CD8+ T cells Cohort 1a Day 15: 0% (placebo) 51% (low dose) 64% (high dose) Cohort 3 0% (placebo) 36% (low dose) 24% (high dose)	[20]
Ad5-nCoV	Mouse	High dose: 5 × 10^9^ vp Middle dose: 5 × 10^8^ vp Low dose: 5 × 10^7^ vp	IM or IN	IM animals: IgG peaked at day 28 NAb titers peaked at week 8 IN animals: IgG peaked week 4 to week 8 NAb titers peaked at week 6 High-dose IN produced higher IgG titers compared to high-dose IM at week 6 and week 8 IM led to a higher ratio of IgG2a to IgG1 compared to IN High-dose IN produced significantly higher NAb titers compared to high-dose IM from week 4 to week 8 Both IM and IN induced S-specific IgG in the trachea-lung but only IN induced S-specific IgA	Middle-dose IM and IN animals showed IFN-γ, TNF-α, and IL-2 responses in splenic CD4+ or CD8+ T cells at week 2 (IM > IN) At week 10, IM induced dose-dependent cellular immunity while IN did not	[22]
	Human	For AI: High dose: 2 × 10^10^ vp Low dose: 1 × 10^10^ vp (day 0 prime + day 28 boost) For IM+AI: 5 × 10^10^ vp IM on day 0 + 2 × 10^10^ vp AI on day 28 For IM: 5 × 10^10^ vp (one dose) or 10 × 10^10^ vp (two doses) on day 0	AI, IM, or both	At day 28 after the last vaccination Neutralizing Ab (GMT): AI high dose: 107 AI low dose: 105 IM+AI: 396 IM one dose: 95 IM two dose: 180 RBD-binding IgG (GMC): AI high dose: 261 AI low dose: 289 IM+AI: 2013 IM one dose: 915 IM two dose: 1190	IFN-γ responses peaked by day 14 after initial immunization for IM and AI vaccinees Aerosol immunization with 1/5 of the IM dose engendered similar IFN-γ responses to that of IM immunizations A boost immunization at day 28 significantly augmented IFN-γ response in the IM+AI group and AI (low dose) group S-specific IFN-γ, IL-2, and TNF-α (but no IL-4) were detected in supernatants of PBMCs 14 days after the first immunization (Th1-biased response)	[32]
hAd5 S-Fusion + N-ETSD	Mouse	1 × 10^10^ vp (SC) 1 × 10^9^ vp (IN)	Combinations of prime and boost through SC and IN	Stronger IgG2a, IgG2b, NAb, and N-specific antibody responses compared to hAd5-S-WT IN prime + IN boost produced similar or better humoral immunity compared to SC prime + SC or IN boost IN + SC prime-only immunization induced similar or better humoral immunity compared to those with a boost	SC prime + SC or IN boost induced stronger T cell responses compared to IN prime + IN boost	[25]
Ad5-S-nb2	Mouse and rhesus macaques	For mouse: 1 × 10^9^ vp IM 5 × 10^9^ vp IM 1 × 10^9^ vp IN 5 × 10^9^ vp IN For macaque: 1 × 10^11^ vp IM 5 × 10^10^ vp IN 1 × 10^10^ vp IM	IM or IN	In mice: 5 × 10^9^ vp IM induced IgGs by day 6 (continued to increase until day 28); 1 × 10^9^ vp IM yielded a lower magnitude of IgG response IN induced weaker IgG responses by day 11 but increased to a similar level by day 28 compared with IM IN induced stronger S-specific IgAs in bronchoalveolar lavage fluids compared to IM In macaques: 1 × 10^11^ vp IM elicited S-specific IgG by day 12 Higher-dose immunizations led to higher IgG titers by day 24 After day 18: IgG continued to increase in IM animals but remained stable in IN animals IM induced 1–2 logs higher serum IgG titers than IN	In both mice and macaques: IM induced stronger systemic cellular immunity than IN	[31]
rAd5 Based (CoroVaxG.3)	Mouse	Single dose of 10^9^ or 10^10^ vp	IM	Induction of S-specific IgG 2 weeks after the single immunization A single immunization of either 109 or 1010 vp induced durable antibody responses for at least 140 days Mouse sera could neutralize pseudo-viruses that expressed D614G, B.1.117, P.1, B.1.617.2 Spikes	Potent IFN-γ-T cell response as early as 2 weeks post- vaccination Durable IFN-γ-T cell response that sustained at a stable level for at least 140 days By day 140, vaccinated animals had central- memory T cells in splenocytes	[62]
Ad5-SARS-CoV-2 spike	Mouse	Prime: 106 PFU (LD) 109 PFU (SD) Boost: 10^9^ PFU	IM	Antibody responses: LD/SD > SD/SD More protracted prime-boost intervals led to better antibody responses	CD8+ T cell response: LD/SD > SD/SD CD4+ T cell response: LD/SD > SD/SD	[23]
ChAdOx1 nCoV-19 (AZD1222)	Human	Prime: 5 × 10^10^ vp (SD) Boost: SD or 2.5 × 10^10^ vp	IM	S-specific IgG (GMT): Day 28: 35,990 Day 56: 25,667 (SD/LD) Day 56: 44,485 (SD/SD) Median NAb titers: Day 28: 451 Day 56: 253 (SD/LD) Day 56: 424 (SD/SD)	IFN-γ-T cell response peaked by day 14 after the initial immunization A boost immunization did not enhance T cell immunity	[63]
	Ferret	Prime or prime boost: 2.5 × 10^10^ vp per dose	IM or IN	Prime-only: IM elicited higher NAbs than IN Prime boost: Both IM and IN induced significantly higher NAb titers 7 days after boost	5 days after SARS-CoV-2 challenge, IM prime-boost animals displayed significantly higher levels of IFN-γ-secreting cells relative to IM prime-only animals	[39]
	Mouse and pig	For mouse: prime or prime boost: 10^8^ IU For pig: prime or prime boost: 10^9^ IU	IM	Mice: Prime-boost animals had stronger binding antibody responses than prime-only animals Pigs: Prime-boost animals had significantly stronger NAb response than prime-only animals (>1-log increase in titer)	Mice: CD4+ and CD8+ T cell responses were similar, irrespective of regimen Pigs: Prime-boost animals exhibited stronger IFN-γ responses relative to prime-only animals 2 weeks post-boost	[37]
	Rhesus macaque	Prime or prime boost: 2.5 × 10^10^ vp	IM	S-specific antibodies significantly increased after boost All prime-boost animals exhibited IgM antibodies Endpoint IgG titers Prime: 400-6400 Prime boost: 400-19,200 NAb titers Prime: 5–40 Prime boost: 10–160	Prime boost elicited similar levels of IFN-γ-T response compared with prime-only	[38]
ChAd-SARS-CoV-2-S	Hamster	Single dose of 10^10^ vp	IM or IN	A single immunization elicited robust S-specific and RBD-specific SARS-CoV-2- neutral izing antibodies IN induced significantly higher antibody levels compared to IM	N/A	[41]
ChAd-SARS-CoV-2-S	Rhesus macaque	Single dose of 10^11^ vp	IN	Day 21: induction of S-specific and RBD-specific binding antibodies Day 21: low levels of IgA present All vaccinated animals developed NAbs NAb titers increased by 10-fold 7 days after challenge compared to 7 days before challenge	All vaccinated animals developed T cell immunity toward the S protein of SARS-CoV-2	[64]
Methyltransferase-defective VSV-based SARS-CoV-2 vaccine (rVSV-D1762A-S)	Mouse and hamster	Mice: 10^6^ PFU Hamsters: 10^6^ PFU	IM	IFNAR1-/-mice (immunocompromised): Developed RBD-specific antibodies that continued to increase from week 2 to week 10 BALB/c mice (immunocompetent): Developed strong NAb responses Hamsters Developed higher levels of NAbs at weeks 4 and 6 compared to convalescent plasma from 10 recovered COVID-19 patients	Th1-biased response	[49]
VSV-based SARS-CoV-2 vaccine (VSV-SARS2-EBOV)	Rhesus macaque	Single dose of 1 × 10^7^ PFU	IM or IN	IM and IN both elicited robust NAb responses	IM elicited stronger cellular immunity compared to IN	[65]
Sputnik V (rAd26-S + rAd5-S)	Human	Prime: rAd26-S: Boost: rAd5-S Formulations: frozen or lyophilized	IM	RBD-specific IgG titers Day 42: 14,703 (frozen) Day 42: l1,143 (lyophilized) NAbs (100% seroconversion) Day 42: 4925 (frozen) Day 42: 4595 (lyophilized)	Cellular response Day 28: 2.5% CD4+ (frozen) Day 28: 1.3% CD8+ (frozen) Day 28: 1.3% CD4+ (lyophilized) Day 28: 1.1% CD8+ (lyophilized)	[35]
Sad23L-nCoV-S-CaP	Mouse	Prime or prime boost: 10^7^ PFU	IM	Elicited strong S-specific antibody responses The boost immunization induced titers of: 105.01 anti-S1 binding Ab 104.77 anti-S2 binding Ab 103.04 pseudo-virus NAb	1466.16 SFCs/10^6^ cells (IFN-γ T cell response to S peptides)	[44,46]
GRAd-COV2	Mouse and macaque	Mice: Single dose of 1 × 10^9^ vp Macaques: Single dose of 5 × 10^10^ vp	IM	Mice: S- and RBD-specific antibodies rapidly rose post-vaccination and increased over time Induction of functional antibodies capable of inhibiting pseudo-type virus Macaques: RBD- and S-specific antibodies peaked between week 2 and week 4 and persisted until at least week 10 Peak NAb titers: 1580–4635 (IC50) NAb titers at week 10 were similar or higher than convalescent titers from recovered COVID-19 patients	Mice: Th1-biased response Macaques: Potent IFN-γ T cell response by week 2 (700–3500 SFC/10^6^ PBMCs) and by week 8 (400–2500 SFC/10^6^ PBMCs) Induction of both CD4+ and CD8+ T cells	[45]
Oncolytic virus (OV-spike)	Mouse	Prime boost (IV): 1 × 106 PFU or 5 × 10^5^ PFU Prime boost (IP): 1 × 106 PFU or 2 × 10^5^ PFU	IV or IP	BALB/c mice: Peak antibody production on day 28 50% of vaccinated mice showed high levels of antibody on day 70 C57BL/6 mice: Peak antibody production on day 21 S-specific antibodies detected as early as day 7 after the first immunization	Induction of CD4+ and CD8+ T cell immunity (ELISPOT: approximately 100 IFNγ+ SFC/ 3 × 10^5^ splenocytes)	[59].
rMeV-preS	Rat, mouse, and hamster	Rats: Day 0: 4 × 10^5^ PFU (SC) Day 28: 2 × 10^6^ PFU Mice: Prime or prime boost Day 0: 8 × 10^5^ PFU (half SC and half IN) Week 4: 8 × 10^5^ PFU Hamsters: Day 0: 8 × 10^5^ PFU (SC and IN) Week 3: 8 × 10^5^ PFU	IN and/or SC	Rats: All vaccinated animals developed S-specific antibodies by week 4 Mice: Prime-boost significantly augmented S-specific antibodies by week 7 compared to prime-only Hamsters: Vaccinated animals developed higher NAb titers at weeks 4 and 6 than those found in sera of 6 convalescent COVID-19 patients	Mice: Th1-biased response	[51]
YF-S0	Hamster, mouse, and macaque	Hamsters: Day 0: 10^3^ PFU (IP) Day 7: boost Mice: Day 0: 400 PFU (IP) Day 7: boost Macaques: Day 0: 10^5^ PFU (SC) Day 7: boost	IP or SC	Hamsters Log-transformed GMT: IgG: 3.5 NAb: 2.2 Mice Log-transformed GMT: IgG: 4.0 NAb: 3.0 Macaques Log-transformed GMT: NAb: 2.6 (day 14) NAb: 2.5 (day 21)	In Mice: Th1-biassed response (ELISPOT: <500 SFC/10^6^ splenocytes)	[55]

Abs: antibodies; AI: aerosol inhalation; GMT: geometric mean titer; GMC: geometric mean concentration; IM: intramuscular route; IN: intranasal route; IP: intraperitoneal route; IU: infectious units; IV: intravenous administration; LOQ: limit of quantitation; LD: low dose; MD: middle dose; HD: high dose; NAb: neutralizing antibody; PFU: plaque forming units; SC: subcutaneous route; SD: standard dose; SFC: spot-forming cell; vp: viral particles.

### 2.2. Cellular Immunity

In addition to their ability to promote antibody production, viral vectors are highly capable of inducing a strong, durable cellular response involving cytotoxic T lymphocytes, which eradicate virally infected cells.

In rhesus macaques, 1 × 10^11^ vp of Ad26.COV2.S elicited strong S-specific T helper 1 (T_H_1)-oriented responses detected by IFN-γ ELISPOT and multiparameter ICS assays 4 weeks post-vaccination [11,19,66]. T_H_1 response was also observed in Ad26.COV2.S-vaccinated mice [17]. Likewise, in a phase 1–2a trial, Ad26.COV2.S induced S-specific T_H_1-oriented CD4+ and CD8+ T cell responses 15 days post-vaccination [20,21]. In a smaller study involving 25 participants, Ad26.COV2.S elicited IFN-γ CD4+ and CD8+ T cell responses that were comparable to those to several SARS-CoV-2 variants (100–200 SFC per 10^6^ PBMC) on day 57 and day 85 post-vaccination [67]. Eight months after a single dose or two doses of Ad26.COV2.S, this same cohort displayed stable CD8+ T cell responses (0.0545% on day 57 vs. 0.0734% on day 239) and slightly dampened CD4+ T cell responses (0.0435% on day 57 vs. 0.0176% on day 239) [68].

For Ad5-nCoV, a single IM immunization in mice prompted IFN-γ, IL-2, and TNFα responses by splenic CD4+ and CD8+ T cells 2 weeks post-vaccination [22]. In a dose-escalation phase 1 trial, Ad5-nCoV was shown to induce T cells as early as 2 weeks after immunization, with a high dose (1.5 × 10^11^ vp) generating higher proportions of polyfunctional T cells than middle (1 × 10^11^ vp) or low (5 × 10^10^ vp) doses [28]. That said, dosage did not significantly affect IFN-γ-ELISPOT responses [27,29]. Intriguingly, aerosolized immunization with one-fifth of the IM dose of Ad5-nCoV induced a similar IFN-γ response to that of IM administrations [32]. While there was a 3–8-fold decrease in T cells from day 14 to day 28, the magnitude of Ad5-nCoV-elicited T cell response on day 28 and day 42 is comparable to that to Ad26.COV2.S on day 57 [27]. Like Ad26.COV2.S, Ad5-nCoV induced a robust T_H_1 response, which was characterized by the secretion of IFN-γ and IL-2 (signature T_H_1 cytokines) [29,32]. Related studies have pinpointed factors such as vaccine formulation and route of immunization that can affect the outcome of T cell response. For example, Rice et al. found that hAd5 S-Fusion + N-ETSD, a dual antigen vaccine comprising the SARS-CoV-2 N and S protein, not only triggered T_H_1 response in mice but also galvanized multifunctional CD4+ and CD8+ T cell responses, which have been shown to correlate with vaccine efficacy [25]. In addition, SC immunization resulted in higher levels of T cell response than IN immunization [25]. As another example, a single immunization of CoroVaxG.3, a chimeric human Ad5-based COVID-19 vaccine, induced robust and durable cellular immunity in mice, which had CD44^high^ CD62L^high^ central-memory T cells in splenocytes 140 days post-vaccination [62].

In a “Sputnik light” study, 96% of volunteers showed IFN-γ T cell responses just 10 days after a single shot of rAd26. The median SFC per 10^6^ PBMCs was 554.3 spots, which is 3-fold higher than that of Ad26.COV2.S [34]. Interestingly, “Sputnik light”, which relies on a prime-only regimen of rAd26, induced similar levels of T cell response in comparison with “Sputnik V”, which utilizes both rAd26 and rAd5 in a heterologous prime-boost approach. [35].

ChAdOx1 nCoV-19 was shown to elicit strong T_H_1-biased responses in mice, pigs, ferrets, and rhesus macaques [37,39]. In macaques, there was no statistically significant difference in the magnitude of response between the prime-only and the prime-boost groups [38]. A similar phenomenon was also observed in humans in which the vaccine regimen did not significantly affect the intensity of response [36,69]. In a phase 1/2 clinical trial, ChAdOx1 nCoV-19 induced polyfunctional cytotoxic CD8+ T cells, IFN-γ- and TNF-α-secreting CD4+ T cells, as well as NK cells [36,40]. IFN-γ responses peaked 14 days after the first shot, and the magnitude of response was similar, irrespective of age and dose [69]. Silva-Cayetano et al. found that a single immunization of ChAdOx1 nCoV-19 elicited S-specific T_H_1 cells, T_H_1-like Foxp3+ regulatory T cells, polyfunctional S-specific CD8+ T cells, and granzyme-B-producing CD8+ T cells in aged and adult mice [70]. This study also showed that a boost immunization in aged mice improved immunogenicity by enhancing the formation of germinal centers and the production of CD8+ effectors [70]. Therefore, compared to prime-only vaccinations, prime-boost vaccination strategies may turn out to produce better immune responses and provide stronger protection for older adults whose immune system function declines with age.

In addition to the VVVs described above, VSV-, measles virus-, and yellow fever virus-based vaccines have also demonstrated the ability to induce T_H_1-oriented responses in various preclinical studies [47,48,49,51,52,55]. Last but not least, the aforementioned OV-spike vaccine was also shown to induce SARS-CoV-2-specific T cell responses in tumor-free and tumor-bearing mice [59].

### 2.3. Innate Immunity

Innate immunity is pivotal in activating adaptive immunity and promoting durable immunological memory. It is well known that the innate immune system, upon exposure to invading microbes, can generate an immediate host defense, featuring the production of inflammatory cytokines and interferons (IFNs), which plays a vital role in the activation, differentiation, and proliferation of T and B cells, effectors of adaptive immunity [71]. Compared with traditional vaccines that require adjuvants to activate innate immunity, many VVVs have “self-adjuvant” properties, as they can spur innate immune signaling cascades through their pathogen-associated molecular patterns (PAMPs), which bind to pattern recognition receptors (PRRs) of host cells [3].

While there have been limited studies investigating the mechanisms of innate immune activation by currently approved COVID-19 vaccines, the overall scheme is as follows, using Ad26.COV2.S as an example (Figure 1). The innate immune system initially interacts with the vaccine at the site of administration. Ad viral particles that encase the double-stranded DNA (dsDNA) genome bump into sentinel cells such as plasmacytoid dendritic cells (pDCs), conventional dendritic cells (DCs), and macrophages, resulting in the intracellular production of high levels of the SARS-CoV-2 S protein [72,73,74]. The adenovirus-cell interaction triggers a series of signaling cascades that galvanizes the production of type I IFNs and various pro-inflammatory cytokines and chemokines, which orchestrate adaptive immune responses. While pDCs stimulate the production of type I IFNs in a toll-like receptor-dependent (TLR-dependent) manner, namely by the TLR9-MyD88 signaling pathway, conventional DCs and macrophages achieve that very objective in a TLR-independent fashion, namely by the cytosolic sensing of adenoviral DNA. At the site of administration, antigen-presenting cells such as DCs can undergo maturation, carry antigen via afferent lymphatic vessels to draining lymph nodes, and prime naïve T cells by presenting them with antigen and co-stimulatory molecules [72]. When these T cells become activated and begin to proliferate, they have the potential to differentiate into memory cells or effectors that comprise cytotoxic CD8+ T cells and helper CD4+ T cells.

There are multiple lines of evidence suggesting that vaccine-induced innate immunity is important for rapidly controlling SARS-CoV-2 infection and shaping downstream adaptive immunity. First, non-specific vaccines such as BCG vaccines and influenza vaccines have been illustrated to reduce COVID-19 disease severity through “innate immune memory” (i.e., trained immunity) [75,76,77,78]. Second, BNT162b2 and mRNA-1273 COVID-19 vaccines were shown to generate a partially protective immune response against symptomatic SARS-CoV-2 infection just days after immunization [79,80]. Third, autoantibodies against type I IFNs have been associated with severe COVID-19 disease, highlighting the vital role of cytokines and innate immune cells in the protection against SARS-CoV-2 [81,82].

### 2.4. Immune Correlates of Protection

In the context of vaccinology, immune correlates of protection inform a statistical relation between immune parameters and clinical outcomes, without necessarily defining causal or mechanistic factor(s). A comprehensive evaluation of correlates of protection from early small immunogenicity-based trials can unveil immune biomarkers that are predictive of vaccine efficacy, from which the vaccine may be subjected to further assessment in large efficacy trials [83].

Data accrued from early human coronavirus studies together with evidence obtained from the COVID-19 outbreak underscore that humoral and cellular immunity are both essential for SARS-CoV-2 clearance [84,85,86,87]. Naturally acquired SARS-CoV-2 immunity has been shown to protect from viral rechallenge in non-human primates [88,89] or reinfection in humans [90,91]. Progress in deciphering biomarker(s) that can be used for predicting COVID-19 disease outcome has led to the finding that antibodies are a key immune signature of protection. For example, the adoptive transfer of purified polyclonal IgG from convalescent rhesus macaques protected naïve recipient macaques from SARS-CoV-2 infection in a dose-dependent manner [92]. To determine the antibody titer thresholds required for protection against SARS-CoV-2 challenge, logistic regression models were employed and revealed that NAb titers of 50, RBD-specific antibody titers of 100, and S-specific antibody titers of 400 were indispensable [92]. In another study, logistic and mechanistic modeling approaches were leveraged and showed that binding antibodies and neutralizing antibodies were linked to increased protection against SARS-CoV-2 replication in the nose and the lung of rhesus macaques, and were predictive of durable protection against virus [93].

With regard to Moderna’s mRNA-1273 COVID-19 vaccine, a phase 3 trial conducted in the US showed that binding and neutralizing antibodies were directly associated with efficacy and inversely associated with disease risk [94]. For ChAdOx1, a phase 2/3 trial conducted in the UK found that higher levels of NAb, anti-RBD IgG, and anti-S IgG were linked to a decreased risk of symptomatic disease [95]. This study also found that 26 IU/ml normalized pseudo-virus NAb titers and 247 normalized live-virus NAb titers were needed to achieve 80% efficacy against symptomatic disease. A comprehensive analysis of the correlation between neutralization level and clinical protection from SARS-CoV-2 using data from convalescent cohorts and seven COVID-19 vaccines further emphasizes the importance of neutralizing antibodies in predicting vaccine efficacy [96]. Nonetheless, it is imperative to standardize neutralization assays and clinical trials in the future, considering that different assays may lead to divergent interpretations about the specific contribution of NAbs to vaccine immunity against SARS-CoV-2.

The significance of T cell immunity in the control of SARS-CoV-2 infection was first demonstrated in non-human primates. In convalescent rhesus macaques, depletion of CD8+ T cells partially abrogated protection against SARS-CoV-2 rechallenge [92], suggesting that T cells are key to combating the virus when antibody levels drop below suboptimal concentrations. Nevertheless, the precise role of cellular immunity in protection against SARS-CoV-2 remains unresolved in macaques vaccinated by a DNA-based vaccine or Ad26.COV2.S [11,18]. Even though currently approved COVID-19 VVVs and mRNA vaccines induce robust T cell responses, the protective threshold of cellular immunity remains to be determined. There is also a number of studies investigating B cell immunity following VVV vaccination. For example, it was shown that rhesus macaques developed RBD-specific IgG+ B cells 2 weeks after immunization with Ad26.COV2.S [19]; S- and RBD-specific memory B cells also correlated with protection against SARS-CoV-2. Additionally, a homologous boost immunization of ChAdOx1 nCoV-19 significantly augmented S-specific memory B cells relative to a prime-only immunization [97]. Moreover, there is evidence supporting that Fc-mediated antibody effector functions can also contribute to protection [11]. Hence, it is important to identify both correlates and mechanisms of protection to navigate the use of different COVID-19 vaccines.

## 3. Durability and Breadth

Ad26.COV2.S is capable of generating potent and relatively durable antibody responses that are not always induced via other vaccine approaches. In rhesus macaques, a single immunization of Ad26.COV2.S elicited robust binding and neutralizing antibody responses that persisted for at least 14 weeks [66]. In humans, a single immunization of Ad26.COV2.S elicited NAbs that maintained at stable levels for at least 8 months and binding antibodies that maintained for at least 6 months [68,98], contrasting with other vaccine modalities [99,100]. Additionally, a single immunization of 10^12^ vp of a thermo-stable adeno-associated viral-based vaccine encoding S (AAVCOVID-1) elicited durable NAb responses that sustained for at least 1 year and protected macaques from SARS-CoV-2 challenge [101].

A single immunization of Ad26.COV2.S increased the breadth of neutralizing antibody coverage against SARS-CoV-2 variants over time [68], which was also observed in convalescent COVID-19 patients [102]. While the mechanisms of durability following Ad26.COV2.S vaccination are yet to be determined, accumulating evidence suggests that prolonged expression of vaccine antigen can facilitate the differentiation of CD8+ T cells into memory populations and stimulate the formation of germinal centers responsible for antibody affinity maturation [103,104,105]. Consequently, memory B cells and long-lived plasma cells are produced, and antibodies can augment their affinity for specific epitopes and expand their breadth of antigen recognition over time.

## 4. Boosters

Waning immunity and emerging SARS-CoV-2 variants will jeopardize vaccine efficacy, particularly in the elderly and in immunosuppressed individuals [106]. Therefore, boosters are becoming more important for strengthening vaccine responses toward SARS-CoV-2.

ChAdOx1 nCoV-19 is one of the most widely employed VVVs for prime-boost vaccinations. In a cohort of 88 healthcare workers who received one shot of ChAdOx1 nCoV-19, a homologous boost immunization 9 to 12 weeks later increased RBD-specific IgG titers by 5-fold and NAb titers by 2-fold; a heterologous boost immunization with mRNA-1273 augmented IgG titers by 125-fold and NAb titers by 20-fold [107]. Importantly, NAbs generated through the heterologous prime-boost regimen were able to neutralize SARS-CoV-2 variants of concern [107].

By virtue of a similar approach, a phase 2 trial conducted in Spain found that a boost immunization of BNT162b2 in company with a prime immunization of ChAdOx1 nCoV-19 increased the production of RBD-specific antibodies by more than 100-fold [108]. In a cohort of 26 clinic employees, those who received heterologous prime-boost vaccinations (ChAdOx1 nCoV-19/BNT162b2) during an 8-week interval exhibited significantly higher levels of IgG, IgA, IgM, NAb, and T cells compared to those who received homologous prime-boost vaccinations (BNT162b2/BNT162b2) [109]. Liu et al. examined the immunogenicity of 4 prime-boost approaches administered 28 days apart: ChAd/ChAd, ChAd/BNT, BNT/BNT, or BNT /ChAd [110]. Compared to ChAd/ChAd, ChAd/BNT resulted in a 9.3-fold and an 8.5-fold increase in S-specific IgGs and NAbs, respectively. Compared to BNT/ChAd, BNT/BNT elicited up to a 2-fold increase in binding and neutralizing antibodies 28 days post-boost vaccination. The strongest humoral immunity is induced by BNT/BNT and ChAd/BNT, followed by BNT/ChAd and ChAd/ChAd. The strongest T cell immunity is induced by ChAd/BNT, followed by BNT/BNT, BNT/ChAd, and ChAd/ChAd [110].

On the same spectrum, Hillus et al. found that heterologous prime-boost regimens were more favorable than homologous prime-boost regimens by assessing the immunogenicity of ChAd/ChAd, ChAd/BNT, and BNT/BNT over a period of 10 to 12 weeks [111]. A phase 2 trial conducted in the UK evaluated the safety and immunogenicity of a third immunization using one of 7 different COVID-19 vaccines after two doses of ChAdOx1 nCoV-19 or BNT162b2 [112]. ChAd/ChAd coupled with a third immunization of mRNA-1273 or BNT162b2 enhanced antibody responses by 20-fold compared to the control, which did not receive a third immunization. CVnCoV, the mRNA vaccine developed by CureVac, in conjunction with Ad26.COV2.S and Novavax’s protein-based vaccine (NVX-CoV2373), augmented antibody titers by 5-fold. ChAd/ChAd plus Valneva’s inactivated whole virus vaccine (VLA2001) enhanced antibody titers by 2-fold. BNT/BNT in conjunction with a third immunization of mRNA-1273 induced the strongest humoral immunity, followed by BNT162b2, Ad26.COV2.S, ChAdOx1, NVX-CoV2373, CVnCoV, and VLA2001 [112].

When primed with Ad26.COV2.S, heterologous boosters with mRNA-1273 or BNT162b2 yielded stronger antibody and T cell responses than homologous boosters [113]. More concretely, Huat et al. showed that in Ad26.COV2.S-vaccinated individuals, heterologous boosters improved the magnitude and breadth of humoral and cellular immune responses [114]. In line with this study, a third immunization of Ad26.COV2.S also improved immunogenicity in 7 individuals who were already vaccinated with two doses of BNT162b2 [115]. Based on the available evidence, longer prime-boost intervals seem to improve vaccine immunogenicity, as studies on Ad26.COV2.S have shown that a later booster (6 months post prime) generated stronger immunity than an earlier booster (2 months post prime) [20,98]. Similar findings were reported for ChAdOx1 nCoV-19 and BNT162b2 [116,117]. In addition, Ad26.COV2.S was shown to be immunogenic in individuals who were immune compromised or did not respond well to mRNA vaccines [118,119]. In all, boosting with VVVs represents a promising approach to enhance immunogenicity, particularly when applied in a heterologous prime-boost regimen.

## 5. Challenges

Despite the clinical success of VVVs, major hurdles remain. For example, pre-existing immunity to vector particles is a major impediment to attaining potent immunogenicity. Since COVID-19 vaccines may become integrated into annual immunization schedules, immunity to the vector could limit its repetitive use.

In a phase 1 trial study, following administration of a recombinant Ad5-vectored COVID-19 vaccine, individuals who had high (>1:200) pre-existing Ad5 NAbs generated lower antibody and T cell responses compared to those with minimal (<1:200) pre-existing Ad5 immunity [28]. In two phase 1/2 trial studies, the magnitude of anti-RBD antibody responses was not affected by the presence of anti-rAd5 and anti-rAd26 antibodies [35]. Additional studies with a larger sample size will be required to decode the impact that pre-existing immunity has on immunogenicity.

Accordingly, massive efforts have been geared toward developing strategies to circumvent vector immunity. For instance, alternative Ad serotypes (human and non-human origin) such as human adenovirus serotype 26 (Ad26.COV2.S) and chimpanzee adenovirus serotype Y25 (ChAdOx1 nCoV-19) have been leveraged to improve immunogenicity. In addition, modifications of the capsid protein hexon, such as the removal of dominant neutralizing antibody epitopes and the replacement of homologous proteins from alternative serotypes, have led to enhanced immunogenicity and vaccine performance [120]. Furthermore, approaches like chemical modifications of adenovirus vectors and complexing Ads with bilamellar cationic liposomes have also shown promise [121,122]. While these strategies could engender potential caveats such as inflammation-related adverse reactions and drastically altered biodistribution profiles, the capacity for intricate molecular manipulations provides exciting opportunities for adenoviral-vectored vaccines to realize their full potential.

Notwithstanding the immunological benefits of VVVs, reports of rare adverse events have begun to surface. One severe side effect following vaccination with adenoviral-vectored vaccines is an extremely rare syndrome called “vaccine-induced immune thrombotic thrombocytopenia” (VITT) [123]. Approximately 1:150,000 ChAdOx1 nCoV-19 recipients and 1:470,000 Ad26.COV2.S recipients have suffered from VITT, which is linked to a mortality rate of 20% to 30% [124]. Intestinal venous and arterial thromboses, cerebral venous sinus thrombosis, as well as thrombocytopenia were observed 5 to 24 days after ChAdOx1 vaccination [125,126]. The precise mechanism(s) underlying vaccine-associated thrombosis and thrombocytopenia, at the time of writing, has not yet been fully elucidated. COVID-19 vaccines that do not use adenovirus vectors were associated with less frequent VITT, and thus some conjectured that VITT complications stemmed directly from the adenovirus itself [127]. In this regard, mechanistic studies showed that the pathogenesis of VITT resembles that of heparin-induced thrombocytopenia [128]. Greinacher et al. demonstrated that the hexon protein could form antigenic complexes with platelet factor 4 (PF4), exposing its neoantigens and provoking the binding of high-avidity anti-PF4 antibodies onto platelet surfaces [128]. Additionally, the contaminants found in vaccines may exacerbate side effects [128]. Moreover, neutrophil extracellular traps may enhance the function of pathologic anti-PF4 antibodies and drive VITT if they form aggregates with PF4 [128]. Based on these findings, one may expect that intravenous (IV) injection has a higher chance of inducing VITT than, say, IM injection. Previous studies in mice demonstrated that IV injection of Ad5 vectors caused platelet activation and aggregation [129]. If VITT is caused by vaccine leakage into blood, alternative approaches such as intranasal immunization or oral vaccine delivery have the potential to bypass this obstacle. Notably, for people who were vaccinated with ChAdOx1, the overall incidence of blood clots was 15.4 cases per million after the first dose and 1.9 cases per million after the second dose. This decrease in incidence hints that vector immunity may mitigate certain vaccine-induced adverse reactions. Besides VITT, rare neurological disorders including acute transverse myelitis, acute disseminated encephalomyelitis, and Guillain–Barré syndrome have also been associated with the ChAdOx1 vaccine [130,131,132,133].

## 6. Conclusions and Future Outlook

As SARS-CoV-2 continues to spread, vaccines remain the most effective tool to save lives until natural immunity or global vaccination is achieved. VVVs are easily stored and administered. Above all, they are incredibly immunogenic, even without the presence of adjuvants. Hence, VVVs hold a strong place in the battleground against COVID-19.

Global access to an array of safe, effective vaccines is crucial to quell the pandemic. Since SARS-CoV-2 is predisposed to infect respiratory mucosa, it is critical to develop vaccines that not only foster systemic immunity but also mucosal immunity to rapidly control viral replication. It is also important to optimize vaccines for protection against symptomatic and ideally asymptomatic infection to decrease the risk of transmission. As the virus evolves and acquires new mutations, it is conceivable that vaccines capable of eliciting antibodies with broad neutralization breadth and T cells that target conserved epitopes will be ideal. Moreover, individual differences in sociodemographic factors and immunocompetence may influence which vaccine best protects a given person.

Achieving consistent safety and high-level immunogenicity requires long-term surveillance of adverse events following vaccination as well as an increased understanding of the immunology of viral vectors. Unfortunately, a lack of trust in vaccine safety and a maelstrom of misinformation about the pandemic have stymied the administration of licensed COVID-19 vaccines worldwide. Therefore, it is paramount to disseminate accessible research findings to the public to increase vaccine acceptance and extinguish the fire that is COVID-19.

## Data Availability

Not applicable.

## References

[1] Coronavirus Disease (COVID-19) Pandemic: Diseases at a Glance. https://www.who.int/emergencies/diseases/novel-coronavirus-2019.

[2] Gebre M.S., Brito L.A., Tostanoski L.H., Edwards D.K., Carfi A., Barouch D.H. (2021). Novel approaches for vaccine development. Cell.

[3] Pinschewer D.D. (2017). Virally vectored vaccine delivery: Medical needs, mechanisms, advantages and challenges. Swiss Med. Wkly..

[4] Draper S.J., Heeney J.L. (2010). Viruses as vaccine vectors for infectious diseases and cancer. Nat. Rev. Microbiol..

[5] (2022). COVID-19 Vaccine Tracker and Landscape. https://www.who.int/publications/m/item/draft-landscape-of-covid-19-candidate-vaccines.

[6] Lundstrom K. (2021). Self-Replicating RNA Viruses for Vaccine Development against Infectious Diseases and Cancer. Vaccines.

[7] Lundstrom K. (2021). Viral Vectors for COVID-19 Vaccine Development. Viruses.

[8] Coughlan L. (2020). Factors Which Contribute to the Immunogenicity of Non-replicating Adenoviral Vectored Vaccines. Front. Immunol..

[9] Lee C.S., Bishop E.S., Zhang R., Yu X., Farina E.M., Yan S., Zhao C., Zheng Z., Shu Y., Wu X. (2017). Adenovirus-Mediated Gene Delivery: Potential Applications for Gene and Cell-Based Therapies in the New Era of Personalized Medicine. Genes Dis..

[10] Tostanoski L.H., Wegmann F., Martinot A.J., Loos C., McMahan K., Mercado N.B., Yu J., Chan C.N., Bondoc S., Starke C.E. (2020). Ad26 vaccine protects against SARS-CoV-2 severe clinical disease in hamsters. Nat. Med..

[11] Mercado N.B., Zahn R., Wegmann F., Loos C., Chandrashekar A., Yu J., Liu J., Peter L., McMahan K., Tostanoski L.H. (2020). Single-shot Ad26 vaccine protects against SARS-CoV-2 in rhesus macaques. Nature.

[12] Mutua G., Anzala O., Luhn K., Robinson C., Bockstal V., Anumendem D., Douoguih M. (2019). Safety and Immunogenicity of a 2-Dose Heterologous Vaccine Regimen With Ad26.ZEBOV and MVA-BN-Filo Ebola Vaccines: 12-Month Data From a Phase 1 Randomized Clinical Trial in Nairobi, Kenya. J. Infect. Dis..

[13] Barouch D.H., Tomaka F.L., Wegmann F., Stieh D.J., Alter G., Robb M.L., Michael N.L., Peter L., Nkolola J.P., Borducchi E.N. (2018). Evaluation of a mosaic HIV-1 vaccine in a multicentre, randomised, double-blind, placebo-controlled, phase 1/2a clinical trial (APPROACH) and in rhesus monkeys (NHP 13-19). Lancet.

[14] Salisch N.C., Stephenson K.E., Williams K., Cox F., van der Fits L., Heerwegh D., Truyers C., Habets M.N., Kanjilal D.G., Larocca R.A. (2021). A Double-Blind, Randomized, Placebo-Controlled Phase 1 Study of Ad26.ZIKV.001, an Ad26-Vectored Anti–Zika Virus Vaccine. Ann. Intern. Med..

[15] Williams K., Bastian A.R., Feldman R.A., Omoruyi E., de Paepe E., Hendriks J., van Zeeburg H., Godeaux O., Langedijk J.P.M., Schuitemaker H. (2020). Phase 1 Safety and Immunogenicity Study of a Respiratory Syncytial Virus Vaccine with an Adenovirus 26 Vector Encoding Prefusion F (Ad26.RSV.preF) in Adults Aged ≥60 Years. J. Infect. Dis..

[16] van der Lubbe J.E.M., Rosendahl Huber S.K., Vijayan A., Dekking L., van Huizen E., Vreugdenhil J., Choi Y., Baert M.R.M., Feddes-de Boer K., Izquierdo Gil A. (2021). Ad26.COV2.S protects Syrian hamsters against G614 spike variant SARS-CoV-2 and does not enhance respiratory disease. NPJ Vaccines.

[17] Bos R., Rutten L., van der Lubbe J.E.M., Bakkers M.J.G., Hardenberg G., Wegmann F., Zuijdgeest D., de Wilde A.H., Koornneef A., Verwilligen A. (2020). Ad26 vector-based COVID-19 vaccine encoding a prefusion-stabilized SARS-CoV-2 Spike immunogen induces potent humoral and cellular immune responses. NPJ Vaccines.

[18] Yu J., Tostanoski Lisa H., Peter L., Mercado Noe B., McMahan K., Mahrokhian Shant H., Nkolola Joseph P., Liu J., Li Z., Chandrashekar A. (2020). DNA vaccine protection against SARS-CoV-2 in rhesus macaques. Science.

[19] He X., Chandrashekar A., Zahn R., Wegmann F., Yu J., Mercado N.B., McMahan K., Martinot A.J., Piedra-Mora C., Beecy S. (2021). Low-dose Ad26.COV2.S protection against SARS-CoV-2 challenge in rhesus macaques. Cell.

[20] Sadoff J., Le Gars M., Shukarev G., Heerwegh D., Truyers C., de Groot A.M., Stoop J., Tete S., Van Damme W., Leroux-Roels I. (2021). Interim Results of a Phase 1-2a Trial of Ad26.COV2.S Covid-19 Vaccine. N. Engl. J. Med..

[21] Stephenson K.E., Le Gars M., Sadoff J., de Groot A.M., Heerwegh D., Truyers C., Atyeo C., Loos C., Chandrashekar A., McMahan K. (2021). Immunogenicity of the Ad26.COV2.S Vaccine for COVID-19. JAMA.

[22] Wu S., Zhong G., Zhang J., Shuai L., Zhang Z., Wen Z., Wang B., Zhao Z., Song X., Chen Y. (2020). A single dose of an adenovirus-vectored vaccine provides protection against SARS-CoV-2 challenge. Nat. Commun..

[23] Sanchez S., Palacio N., Dangi T., Ciucci T., Penaloza-MacMaster P. (2021). Fractionating a COVID-19 Ad5-vectored vaccine improves virus-specific immunity. Sci. Immunol..

[24] Knoll M.D., Wonodi C. (2021). Oxford–AstraZeneca COVID-19 vaccine efficacy. Lancet.

[25] Rice A., Verma M., Shin A., Zakin L., Sieling P., Tanaka S., Balint J., Dinkins K., Adisetiyo H., Morimoto B. (2021). Intranasal plus subcutaneous prime vaccination with a dual antigen COVID-19 vaccine elicits T-cell and antibody responses in mice. Sci. Rep..

[26] Gabitzsch E., Safrit J.T., Verma M., Rice A., Sieling P., Zakin L., Shin A., Morimoto B., Adisetiyo H., Wong R. (2021). Dual-Antigen COVID-19 Vaccine Subcutaneous Prime Delivery with Oral Boosts Protects NHP Against SARS-CoV-2 Challenge. Front. Immunol..

[27] Zhu F.C., Guan X.H., Li Y.H., Huang J.Y., Jiang T., Hou L.H., Li J.X., Yang B.F., Wang L., Wang W.J. (2020). Immunogenicity and safety of a recombinant adenovirus type-5-vectored COVID-19 vaccine in healthy adults aged 18 years or older: A randomised, double-blind, placebo-controlled, phase 2 trial. Lancet.

[28] Zhu F.C., Li Y.H., Guan X.H., Hou L.H., Wang W.J., Li J.X., Wu S.P., Wang B.S., Wang Z., Wang L. (2020). Safety, tolerability, and immunogenicity of a recombinant adenovirus type-5 vectored COVID-19 vaccine: A dose-escalation, open-label, non-randomised, first-in-human trial. Lancet.

[29] Zhu F., Jin P., Zhu T., Wang W., Ye H., Pan H., Hou L., Li J., Wang X., Wu S. (2021). Safety and immunogenicity of a recombinant adenovirus type-5-vectored COVID-19 vaccine with a homologous prime-boost regimen in healthy participants aged 6 years and above: A randomised, double-blind, placebo-controlled, phase 2b trial. Clin. Infect. Dis. Off. Publ. Infect. Dis. Soc. Am..

[30] Kim E., Weisel F.J., Balmert S.C., Khan M.S., Huang S., Erdos G., Kenniston T.W., Carey C.D., Joachim S.M., Conter L.J. (2021). A single subcutaneous or intranasal immunization with adenovirus-based SARS-CoV-2 vaccine induces robust humoral and cellular immune responses in mice. Eur. J. Immunol..

[31] Feng L., Wang Q., Shan C., Yang C., Feng Y., Wu J., Liu X., Zhou Y., Jiang R., Hu P. (2020). An adenovirus-vectored COVID-19 vaccine confers protection from SARS-COV-2 challenge in rhesus macaques. Nat. Commun..

[32] Wu S., Huang J., Zhang Z., Wu J., Zhang J., Hu H., Zhu T., Zhang J., Luo L., Fan P. (2021). Safety, tolerability, and immunogenicity of an aerosolised adenovirus type-5 vector-based COVID-19 vaccine (Ad5-nCoV) in adults: Preliminary report of an open-label and randomised phase 1 clinical trial. Lancet. Infect. Dis..

[33] Jones I., Roy P. (2021). Sputnik V COVID-19 vaccine candidate appears safe and effective. Lancet.

[34] Tukhvatulin A.I., Dolzhikova I.V., Shcheblyakov D.V., Zubkova O.V., Dzharullaeva A.S., Kovyrshina A.V., Lubenets N.L., Grousova D.M., Erokhova A.S., Botikov A.G. (2021). An open, non-randomised, phase 1/2 trial on the safety, tolerability, and immunogenicity of single-dose vaccine “Sputnik Light” for prevention of coronavirus infection in healthy adults. Lancet Reg. Health. Eur..

[35] Logunov D.Y., Dolzhikova I.V., Zubkova O.V., Tukhvatullin A.I., Shcheblyakov D.V., Dzharullaeva A.S., Grousova D.M., Erokhova A.S., Kovyrshina A.V., Botikov A.G. (2020). Safety and immunogenicity of an rAd26 and rAd5 vector-based heterologous prime-boost COVID-19 vaccine in two formulations: Two open, non-randomised phase 1/2 studies from Russia. Lancet.

[36] Folegatti P.M., Ewer K.J., Aley P.K., Angus B., Becker S., Belij-Rammerstorfer S., Bellamy D., Bibi S., Bittaye M., Clutterbuck E.A. (2020). Safety and immunogenicity of the ChAdOx1 nCoV-19 vaccine against SARS-CoV-2: A preliminary report of a phase 1/2, single-blind, randomised controlled trial. Lancet.

[37] Graham S.P., McLean R.K., Spencer A.J., Belij-Rammerstorfer S., Wright D., Ulaszewska M., Edwards J.C., Hayes J.W.P., Martini V., Thakur N. (2020). Evaluation of the immunogenicity of prime-boost vaccination with the replication-deficient viral vectored COVID-19 vaccine candidate ChAdOx1 nCoV-19. NPJ Vaccines.

[38] van Doremalen N., Lambe T., Spencer A., Belij-Rammerstorfer S., Purushotham J.N., Port J.R., Avanzato V.A., Bushmaker T., Flaxman A., Ulaszewska M. (2020). ChAdOx1 nCoV-19 vaccine prevents SARS-CoV-2 pneumonia in rhesus macaques. Nature.

[39] Marsh G.A., McAuley A.J., Au G.G., Riddell S., Layton D., Singanallur N.B., Layton R., Payne J., Durr P.A., Bender H. (2021). ChAdOx1 nCoV-19 (AZD1222) vaccine candidate significantly reduces SARS-CoV-2 shedding in ferrets. NPJ Vaccines.

[40] Ewer K.J., Barrett J.R., Belij-Rammerstorfer S., Sharpe H., Makinson R., Morter R., Flaxman A., Wright D., Bellamy D., Bittaye M. (2021). T cell and antibody responses induced by a single dose of ChAdOx1 nCoV-19 (AZD1222) vaccine in a phase 1/2 clinical trial. Nat. Med..

[41] Bricker T.L., Darling T.L., Hassan A.O., Harastani H.H., Soung A., Jiang X., Dai Y.N., Zhao H., Adams L.J., Holtzman M.J. (2021). A single intranasal or intramuscular immunization with chimpanzee adenovirus-vectored SARS-CoV-2 vaccine protects against pneumonia in hamsters. Cell Rep..

[42] Hassan A.O., Kafai N.M., Dmitriev I.P., Fox J.M., Smith B.K., Harvey I.B., Chen R.E., Winkler E.S., Wessel A.W., Case J.B. (2020). A Single-Dose Intranasal ChAd Vaccine Protects Upper and Lower Respiratory Tracts against SARS-CoV-2. Cell.

[43] Hassan A.O., Shrihari S., Gorman M.J., Ying B., Yaun D., Raju S., Chen R.E., Dmitriev I.P., Kashentseva E., Adams L.J. (2021). An intranasal vaccine durably protects against SARS-CoV-2 variants in mice. Cell Rep..

[44] Luo S., Zhang P., Zou P., Wang C., Liu B., Wu C., Li T., Zhang L., Zhang Y., Li C. (2021). A Self-Biomineralized Novel Adenovirus Vectored COVID-19 Vaccine for Boosting Immunization of Mice. Virol. Sin..

[45] Capone S., Raggioli A., Gentile M., Battella S., Lahm A., Sommella A., Contino A.M., Urbanowicz R.A., Scala R., Barra F. (2021). Immunogenicity of a new gorilla adenovirus vaccine candidate for COVID-19. Mol. Ther. J. Am. Soc. Gene Ther..

[46] Luo S., Zhang P., Liu B., Yang C., Liang C., Wang Q., Zhang L., Tang X., Li J., Hou S. (2021). Prime-boost vaccination of mice and rhesus macaques with two novel adenovirus vectored COVID-19 vaccine candidates. Emerg. Microbes Infect..

[47] Malherbe D.C., Kurup D., Wirblich C., Ronk A.J., Mire C., Kuzmina N., Shaik N., Periasamy S., Hyde M.A., Williams J.M. (2021). A single dose of replication-competent VSV-vectored vaccine expressing SARS-CoV-2 S1 protects against virus replication in a hamster model of severe COVID-19. NPJ Vaccines.

[48] Yahalom-Ronen Y., Tamir H., Melamed S., Politi B., Shifman O., Achdout H., Vitner E.B., Israeli O., Milrot E., Stein D. (2020). A single dose of recombinant VSV-G-spike vaccine provides protection against SARS-CoV-2 challenge. Nat. Commun..

[49] Lu M., Zhang Y., Dravid P., Li A., Zeng C., Kc M., Trivedi S., Sharma H., Chaiwatpongsakorn S., Zani A. (2021). A Methyltransferase-Defective Vesicular Stomatitis Virus-Based SARS-CoV-2 Vaccine Candidate Provides Complete Protection against SARS-CoV-2 Infection in Hamsters. J. Virol..

[50] Case J.B., Rothlauf P.W., Chen R.E., Kafai N.M., Fox J.M., Smith B.K., Shrihari S., McCune B.T., Harvey I.B., Keeler S.P. (2020). Replication-Competent Vesicular Stomatitis Virus Vaccine Vector Protects against SARS-CoV-2-Mediated Pathogenesis in Mice. Cell Host Microbe.

[51] Lu M., Dravid P., Zhang Y., Trivedi S., Li A., Harder O., Kc M., Chaiwatpongsakorn S., Zani A., Kenney A. (2021). A safe and highly efficacious measles virus-based vaccine expressing SARS-CoV-2 stabilized prefusion spike. Proc. Natl. Acad. Sci. USA.

[52] Frantz P.N., Barinov A., Ruffie C., Combredet C., Najburg V., de Melo G.D., Larrous F., Kergoat L., Teeravechyan S., Jongkaewwattana A. (2021). A live measles-vectored COVID-19 vaccine induces strong immunity and protection from SARS-CoV-2 challenge in mice and hamsters. Nat. Commun..

[53] Merck and IAVI Discontinue Development of COVID-19 Vaccine Candidate V590. https://www.iavi.org/news-resources/press-releases/2021/merck-and-iavi-discontinue-development-of-covid-19-vaccine-candidate-v590.

[54] Merck Discontinues Development of SARS-CoV-2/COVID-19 Vaccine Candidates; Continues Development of Two Investigational Therapeutic Candidates. https://www.merck.com/news/merck-discontinues-development-of-sars-cov-2-covid-19-vaccine-candidates-continues-development-of-two-investigational-therapeutic-candidates/.

[55] Sanchez-Felipe L., Vercruysse T., Sharma S., Ma J., Lemmens V., Van Looveren D., Arkalagud Javarappa M.P., Boudewijns R., Malengier-Devlies B., Liesenborghs L. (2021). A single-dose live-attenuated YF17D-vectored SARS-CoV-2 vaccine candidate. Nature.

[56] An D., Li K., Rowe D.K., Diaz M.C.H., Griffin E.F., Beavis A.C., Johnson S.K., Padykula I., Jones C.A., Briggs K. (2021). Protection of K18-hACE2 mice and ferrets against SARS-CoV-2 challenge by a single-dose mucosal immunization with a parainfluenza virus 5-based COVID-19 vaccine. Sci. Adv..

[57] Kurup D., Malherbe D.C., Wirblich C., Lambert R., Ronk A.J., Zabihi Diba L., Bukreyev A., Schnell M.J. (2021). Inactivated rabies virus vectored SARS-CoV-2 vaccine prevents disease in a Syrian hamster model. PLoS Pathog..

[58] Sun W., Leist S.R., McCroskery S., Liu Y., Slamanig S., Oliva J., Amanat F., Schafer A., Dinnon K.H., Garcia-Sastre A. (2020). Newcastle disease virus (NDV) expressing the spike protein of SARS-CoV-2 as a live virus vaccine candidate. EBioMedicine.

[59] Sun Y., Dong W., Tian L., Rao Y., Qin C., Jaramillo S.A., Settles E.W., Ma S., Zhang J., Yu K. (2021). Dual roles of a novel oncolytic viral vector-based SARS-CoV-2 vaccine: Preventing COVID-19 and treating tumor progression. Biorxiv Prepr. Serv. Biol..

[60] Liu R., Americo J.L., Cotter C.A., Earl P.L., Erez N., Peng C., Moss B. (2021). One or two injections of MVA-vectored vaccine shields hACE2 transgenic mice from SARS-CoV-2 upper and lower respiratory tract infection. Proc. Natl. Acad. Sci. USA.

[61] Tscherne A., Schwarz J.H., Rohde C., Kupke A., Kalodimou G., Limpinsel L., Okba N.M.A., Bosnjak B., Sandrock I., Odak I. (2021). Immunogenicity and efficacy of the COVID-19 candidate vector vaccine MVA-SARS-2-S in preclinical vaccination. Proc. Natl. Acad. Sci. USA.

[62] Lopez M.V., Vinzon S.E., Cafferata E.G.A., Nunez F.J., Soto A., Sanchez-Lamas M., Afonso M.J., Aguilar-Cortes D., Rios G.D., Maricato J.T. (2021). A Single Dose of a Hybrid hAdV5-Based Anti-COVID-19 Vaccine Induces a Long-Lasting Immune Response and Broad Coverage against VOC. Vaccines.

[63] Barrett J.R., Belij-Rammerstorfer S., Dold C., Ewer K.J., Folegatti P.M., Gilbride C., Halkerston R., Hill J., Jenkin D., Stockdale L. (2021). Phase 1/2 trial of SARS-CoV-2 vaccine ChAdOx1 nCoV-19 with a booster dose induces multifunctional antibody responses. Nat. Med..

[64] Hassan A.O., Feldmann F., Zhao H., Curiel D.T., Okumura A., Tang-Huau T.-L., Case J.B., Meade-White K., Callison J., Chen R.E. (2021). A single intranasal dose of chimpanzee adenovirus-vectored vaccine protects against SARS-CoV-2 infection in rhesus macaques. Cell Rep. Med..

[65] Furuyama W., Shifflett K., Pinski A.N., Griffin A.J., Feldmann F., Okumura A., Gourdine T., Jankeel A., Lovaglio J., Hanley P.W. (2021). Rapid protection from COVID-19 in nonhuman primates vaccinated intramuscularly but not intranasally with a single dose of a recombinant vaccine. Biorxiv Prepr. Serv. Biol..

[66] Solforosi L., Kuipers H., Jongeneelen M., Rosendahl Huber S.K., van der Lubbe J.E.M., Dekking L., Czapska-Casey D.N., Izquierdo Gil A., Baert M.R.M., Drijver J. (2021). Immunogenicity and efficacy of one and two doses of Ad26.COV2.S COVID vaccine in adult and aged NHP. J. Exp. Med..

[67] Alter G., Yu J., Liu J., Chandrashekar A., Borducchi E.N., Tostanoski L.H., McMahan K., Jacob-Dolan C., Martinez D.R., Chang A. (2021). Immunogenicity of Ad26.COV2.S vaccine against SARS-CoV-2 variants in humans. Nature.

[68] Barouch D.H., Stephenson K.E., Sadoff J., Yu J., Chang A., Gebre M., McMahan K., Liu J., Chandrashekar A., Patel S. (2021). Durable Humoral and Cellular Immune Responses 8 Months after Ad26.COV2.S Vaccination. N. Engl. J. Med..

[69] Ramasamy M.N., Minassian A.M., Ewer K.J., Flaxman A.L., Folegatti P.M., Owens D.R., Voysey M., Aley P.K., Angus B., Babbage G. (2021). Safety and immunogenicity of ChAdOx1 nCoV-19 vaccine administered in a prime-boost regimen in young and old adults (COV002): A single-blind, randomised, controlled, phase 2/3 trial. Lancet.

[70] Silva-Cayetano A., Foster W.S., Innocentin S., Belij-Rammerstorfer S., Spencer A.J., Burton O.T., Fra-Bido S., Le Lee J., Thakur N., Conceicao C. (2021). A booster dose enhances immunogenicity of the COVID-19 vaccine candidate ChAdOx1 nCoV-19 in aged mice. Med.

[71] McNab F., Mayer-Barber K., Sher A., Wack A., O’Garra A. (2015). Type I interferons in infectious disease. Nat. Rev. Immunol..

[72] Teijaro J.R., Farber D.L. (2021). COVID-19 vaccines: Modes of immune activation and future challenges. Nat. Rev. Immunol..

[73] Zhu J., Huang X., Yang Y. (2007). Innate Immune Response to Adenoviral Vectors Is Mediated by both Toll-Like Receptor-Dependent and -Independent Pathways. J. Virol..

[74] Fejer G., Freudenberg M., Greber U.F., Gyory I. (2011). Adenovirus-triggered innate signalling pathways. Eur. J. Microbiol. Immunol..

[75] Mantovani A., Netea M.G. (2020). Trained Innate Immunity, Epigenetics, and Covid-19. N. Engl. J. Med..

[76] Escobar L.E., Molina-Cruz A., Barillas-Mury C. (2020). BCG vaccine protection from severe coronavirus disease 2019 (COVID-19). Proc. Natl. Acad. Sci. USA.

[77] Rivas M.N., Ebinger J.E., Wu M., Sun N., Braun J., Sobhani K., Van Eyk J.E., Cheng S., Arditi M. (2021). BCG vaccination history associates with decreased SARS-CoV-2 seroprevalence across a diverse cohort of health care workers. J. Clin. Investig..

[78] Debisarun P.A., Gössling K.L., Bulut O., Kilic G., Zoodsma M., Liu Z., Oldenburg M., Rüchel N., Zhang B., Xu C.-J. (2021). Induction of trained immunity by influenza vaccination—Impact on COVID-19. PLoS Pathog..

[79] Polack F.P., Thomas S.J., Kitchin N., Absalon J., Gurtman A., Lockhart S., Perez J.L., Pérez Marc G., Moreira E.D., Zerbini C. (2020). Safety and Efficacy of the BNT162b2 mRNA Covid-19 Vaccine. N. Engl. J. Med..

[80] Baden L.R., El Sahly H.M., Essink B., Kotloff K., Frey S., Novak R., Diemert D., Spector S.A., Rouphael N., Creech C.B. (2020). Efficacy and Safety of the mRNA-1273 SARS-CoV-2 Vaccine. N. Engl. J. Med..

[81] Bastard P., Gervais A., Le Voyer T., Rosain J., Philippot Q., Manry J., Michailidis E., Hoffmann H.-H., Eto S., Garcia-Prat M. (2021). Autoantibodies neutralizing type I IFNs are present in ~4% of uninfected individuals over 70 years old and account for ~20% of COVID-19 deaths. Sci. Immunol..

[82] Bastard P., Rosen Lindsey B., Zhang Q., Michailidis E., Hoffmann H.-H., Zhang Y., Dorgham K., Philippot Q., Rosain J., Béziat V. (2020). Autoantibodies against type I IFNs in patients with life-threatening COVID-19. Science.

[83] Krammer F. (2021). Correlates of protection from SARS-CoV-2 infection. Lancet.

[84] Bradburne A.F., Bynoe M.L., Tyrrell D.A. (1967). Effects of a “New” Human Respiratory Virus in Volunteers. Br. Med. J..

[85] Barrow G.I., Higgins P.G., Al-Nakib W., Smith A.P., Wenham R.B.M., Tyrrell D.A.J. (1990). The effect of intranasal nedocromil sodium on viral upper respiratory tract infections in human volunteers. Clin. Exp. Allergy.

[86] Cheng Y., Wong R., Soo Y.O.Y., Wong W.S., Lee C.K., Ng M.H.L., Chan P., Wong K.C., Leung C.B., Cheng G. (2005). Use of convalescent plasma therapy in SARS patients in Hong Kong. Eur. J. Clin. Microbiol. Infect. Dis..

[87] Zhao J., Zhao J., Perlman S. (2010). T Cell Responses Are Required for Protection from Clinical Disease and for Virus Clearance in Severe Acute Respiratory Syndrome Coronavirus-Infected Mice. J. Virol..

[88] Chandrashekar A., Liu J., Martinot A.J., McMahan K., Mercado N.B., Peter L., Tostanoski L.H., Yu J., Maliga Z., Nekorchuk M. (2020). SARS-CoV-2 infection protects against rechallenge in rhesus macaques. Science.

[89] Deng W., Bao L., Liu J., Xiao C., Liu J., Xue J., Lv Q., Qi F., Gao H., Yu P. (2020). Primary exposure to SARS-CoV-2 protects against reinfection in rhesus macaques. Science.

[90] Hall V.J., Foulkes S., Charlett A., Atti A., Monk E.J.M., Simmons R., Wellington E., Cole M.J., Saei A., Oguti B. (2021). SARS-CoV-2 infection rates of antibody-positive compared with antibody-negative health-care workers in England: A large, multicentre, prospective cohort study (SIREN). Lancet.

[91] Addetia A., Crawford Katharine H.D., Dingens A., Zhu H., Roychoudhury P., Huang M.-L., Jerome Keith R., Bloom Jesse D., Greninger Alexander L., McAdam Alexander J. (2020). Neutralizing Antibodies Correlate with Protection from SARS-CoV-2 in Humans during a Fishery Vessel Outbreak with a High Attack Rate. J. Clin. Microbiol..

[92] McMahan K., Yu J., Mercado N.B., Loos C., Tostanoski L.H., Chandrashekar A., Liu J., Peter L., Atyeo C., Zhu A. (2021). Correlates of protection against SARS-CoV-2 in rhesus macaques. Nature.

[93] Roozendaal R., Solforosi L., Stieh D.J., Serroyen J., Straetemans R., Dari A., Boulton M., Wegmann F., Rosendahl Huber S.K., van der Lubbe J.E.M. (2021). SARS-CoV-2 binding and neutralizing antibody levels after Ad26.COV2.S vaccination predict durable protection in rhesus macaques. Nat. Commun..

[94] Gilbert P.B., Montefiori D.C., McDermott A.B., Fong Y., Benkeser D., Deng W., Zhou H., Houchens C.R., Martins K., Jayashankar L. (2021). Immune correlates analysis of the mRNA-1273 COVID-19 vaccine efficacy clinical trial. Science.

[95] Feng S., Phillips D.J., White T., Sayal H., Aley P.K., Bibi S., Dold C., Fuskova M., Gilbert S.C., Hirsch I. (2021). Correlates of protection against symptomatic and asymptomatic SARS-CoV-2 infection. Nat. Med..

[96] Khoury D.S., Cromer D., Reynaldi A., Schlub T.E., Wheatley A.K., Juno J.A., Subbarao K., Kent S.J., Triccas J.A., Davenport M.P. (2021). Neutralizing antibody levels are highly predictive of immune protection from symptomatic SARS-CoV-2 infection. Nat. Med..

[97] Barros-Martins J., Hammerschmidt S.I., Cossmann A., Odak I., Stankov M.V., Morillas Ramos G., Dopfer-Jablonka A., Heidemann A., Ritter C., Friedrichsen M. (2021). Immune responses against SARS-CoV-2 variants after heterologous and homologous ChAdOx1 nCoV-19/BNT162b2 vaccination. Nat. Med..

[98] Sadoff J., Le Gars M., Cardenas V., Shukarev G., Vaissiere N., Heerwegh D., Truyers C., de Groot A.M., Scheper G., Hendriks J. (2021). Durability of antibody responses elicited by a single dose of Ad26.COV2.S and substantial increase following late boosting. Medrxiv Prepr. Serv. Health Sci..

[99] Pegu A., O’Connell Sarah E., Schmidt Stephen D., O’Dell S., Talana Chloe A., Lai L., Albert J., Anderson E., Bennett H., Corbett Kizzmekia S. (2021). Durability of mRNA-1273 vaccine–induced antibodies against SARS-CoV-2 variants. Science.

[100] Doria-Rose N., Suthar M.S., Makowski M., O’Connell S., McDermott A.B., Flach B., Ledgerwood J.E., Mascola J.R., Graham B.S., Lin B.C. (2021). Antibody Persistence through 6 Months after the Second Dose of mRNA-1273 Vaccine for Covid-19. N. Engl. J. Med..

[101] Zabaleta N., Dai W., Bhatt U., Herate C., Maisonnasse P., Chichester J.A., Sanmiguel J., Estelien R., Michalson K.T., Diop C. (2021). An AAV-based, room-temperature-stable, single-dose COVID-19 vaccine provides durable immunogenicity and protection in non-human primates. Cell Host Microbe.

[102] Wang Z., Muecksch F., Schaefer-Babajew D., Finkin S., Viant C., Gaebler C., Hoffmann H.-H., Barnes C.O., Cipolla M., Ramos V. (2021). Naturally enhanced neutralizing breadth against SARS-CoV-2 one year after infection. Nature.

[103] Tatsis N., Fitzgerald J.C., Reyes-Sandoval A., Harris-McCoy K.C., Hensley S.E., Zhou D., Lin S.-W., Bian A., Xiang Z.Q., Iparraguirre A. (2007). Adenoviral vectors persist in vivo and maintain activated CD8+ T cells: Implications for their use as vaccines. Blood.

[104] Muecksch F., Weisblum Y., Barnes C.O., Schmidt F., Schaefer-Babajew D., Wang Z., Lorenzi J.C., Flyak A.I., DeLaitsch A.T., Huey-Tubman K.E. (2021). Affinity maturation of SARS-CoV-2 neutralizing antibodies confers potency, breadth, and resilience to viral escape mutations. Immunity.

[105] Gaebler C., Wang Z., Lorenzi J.C.C., Muecksch F., Finkin S., Tokuyama M., Cho A., Jankovic M., Schaefer-Babajew D., Oliveira T.Y. (2021). Evolution of antibody immunity to SARS-CoV-2. Nature.

[106] Goldberg Y., Mandel M., Bar-On Y.M., Bodenheimer O., Freedman L., Haas E.J., Milo R., Alroy-Preis S., Ash N., Huppert A. (2021). Waning Immunity after the BNT162b2 Vaccine in Israel. N. Engl. J. Med..

[107] Normark J., Vikstrom L., Gwon Y.D., Persson I.L., Edin A., Bjorsell T., Dernstedt A., Christ W., Tevell S., Evander M. (2021). Heterologous ChAdOx1 nCoV-19 and mRNA-1273 Vaccination. N. Engl. J. Med..

[108] Borobia A.M., Carcas A.J., Pérez-Olmeda M., Castaño L., Bertran M.J., García-Pérez J., Campins M., Portolés A., González-Pérez M., García Morales M.T. (2021). Immunogenicity and reactogenicity of BNT162b2 booster in ChAdOx1-S-primed participants (CombiVacS): A multicentre, open-label, randomised, controlled, phase 2 trial. Lancet.

[109] Groß R., Zanoni M., Seidel A., Conzelmann C., Gilg A., Krnavek D., Erdemci-Evin S., Mayer B., Hoffmann M., Pöhlmann S. (2022). Heterologous ChAdOx1 nCoV-19 and BNT162b2 prime-boost vaccination elicits potent neutralizing antibody responses and T cell reactivity against prevalent SARS-CoV-2 variants. EBioMedicine.

[110] Liu X., Shaw R.H., Stuart A.S.V., Greenland M., Aley P.K., Andrews N.J., Cameron J.C., Charlton S., Clutterbuck E.A., Collins A.M. (2021). Safety and immunogenicity of heterologous versus homologous prime-boost schedules with an adenoviral vectored and mRNA COVID-19 vaccine (Com-COV): A single-blind, randomised, non-inferiority trial. Lancet.

[111] Hillus D., Schwarz T., Tober-Lau P., Vanshylla K., Hastor H., Thibeault C., Jentzsch S., Helbig E.T., Lippert L.J., Tscheak P. (2021). Safety, reactogenicity, and immunogenicity of homologous and heterologous prime-boost immunisation with ChAdOx1 nCoV-19 and BNT162b2: A prospective cohort study. Lancet Respir. Med..

[112] Munro A.P.S., Janani L., Cornelius V., Aley P.K., Babbage G., Baxter D., Bula M., Cathie K., Chatterjee K., Dodd K. (2021). Safety and immunogenicity of seven COVID-19 vaccines as a third dose (booster) following two doses of ChAdOx1 nCov-19 or BNT162b2 in the UK (COV-BOOST): A blinded, multicentre, randomised, controlled, phase 2 trial. Lancet.

[113] Sablerolles R.S.G., Rietdijk W.J.R., Goorhuis A., Postma D.F., Visser L.G., Geers D., Schmitz K.S., Garrido H.M.G., Koopmans M.P.G., Dalm V.A.S.H. (2021). Immunogenicity and reactogenicity of booster vaccinations after Ad26.COV2.S priming. MedRxiv.

[114] Kim Huat N.K., Er Lim J.M., Gill U.S., de Alwis R., Tan N., Nan Toh J.Z., Abbott J.E., Usai C., Ooi E.E., Hong Low J.G. (2021). Differential immunogenicity of homologous versus heterologous boost in Ad26.COV2.S vaccine recipients. MedRxiv.

[115] Iketani S., Liu L., Nair M.S., Chandrashekar A., Mohri H., Wang M., Barouch D.H., Huang Y., Ho D.D. (2021). Ad26.COV2.S boosts antibody and T-cell responses following BNT162b2 vaccination. Emerg. Microbes Infect..

[116] Voysey M., Costa Clemens S.A., Madhi S.A., Weckx L.Y., Folegatti P.M., Aley P.K., Angus B., Baillie V.L., Barnabas S.L., Bhorat Q.E. (2021). Single-dose administration and the influence of the timing of the booster dose on immunogenicity and efficacy of ChAdOx1 nCoV-19 (AZD1222) vaccine: A pooled analysis of four randomised trials. Lancet.

[117] Payne R.P., Longet S., Austin J.A., Skelly D.T., Dejnirattisai W., Adele S., Meardon N., Faustini S., Al-Taei S., Moore S.C. (2021). Immunogenicity of standard and extended dosing intervals of BNT162b2 mRNA vaccine. Cell.

[118] Reimann P., Ulmer H., Mutschlechner B., Benda M., Severgnini L., Volgger A., Lang T., Atzl M., Huynh M., Gasser K. (2022). Efficacy and safety of heterologous booster vaccination with Ad26.COV2.S after BNT162b2 mRNA COVID-19 vaccine in haemato-oncological patients with no antibody response. Br. J. Haematol..

[119] Khan K., Lustig G., Bernstein M., Archary D., Cele S., Karim F., Smith M., Ganga Y., Jule Z., Reedoy K. (2021). Immunogenicity of SARS-CoV-2 infection and Ad26.CoV2.S vaccination in people living with HIV. Clin. Infect. Dis..

[120] Roberts D.M., Nanda A., Havenga M.J.E., Abbink P., Lynch D.M., Ewald B.A., Liu J., Thorner A.R., Swanson P.E., Gorgone D.A. (2006). Hexon-chimaeric adenovirus serotype 5 vectors circumvent pre-existing anti-vector immunity. Nature.

[121] Suzuki-Kouyama E., Katayama K., Sakurai F., Yamaguchi T., Kurachi S., Kawabata K., Nakagawa S., Mizuguchi H. (2011). Hexon-specific PEGylated adenovirus vectors utilizing avidin-biotin interaction. Biomaterials.

[122] Yotnda P., Chen D.-H., Chiu W., Piedra P.A., Davis A., Templeton N.S., Brenner M.K. (2002). Bilamellar Cationic Liposomes Protect Adenovectors from Preexisting Humoral Immune Responses. Mol. Ther..

[123] Rodriguez E.V.C., Bouazza F.Z., Dauby N., Mullier F., d’Otreppe S., Jissendi Tchofo P., Bartiaux M., Sirjacques C., Roman A., Hermans C. (2021). Fatal vaccine-induced immune thrombotic thrombocytopenia (VITT) post Ad26.COV2.S: First documented case outside US. Infection.

[124] Siegler J.E., Klein P., Yaghi S., Vigilante N., Abdalkader M., Coutinho J.M., Abdul Khalek F., Nguyen T.N. (2021). Cerebral Vein Thrombosis With Vaccine-Induced Immune Thrombotic Thrombocytopenia. Stroke.

[125] Gurtler L., Seitz R., Schramm W. (2021). Cerebral venous thrombosis after COVID-19 vaccination: Is the risk of thrombosis increased by intravascular application of the vaccine?. Infection.

[126] Gaunt E.R., Mabbott N.A. (2021). The clinical correlates of vaccine-induced immune thrombotic thrombocytopenia after immunisation with adenovirus vector-based SARS-CoV-2 vaccines. Immunother. Adv..

[127] Monagle P., Ng A.P., Linden M., Ignjatovic V., Farley A., Taoudi S., Pasricha S.R., Torresi J. (2021). Vaccine-induced immune thrombosis and thrombocytopenia syndrome following adenovirus-vectored severe acute respiratory syndrome coronavirus 2 vaccination: A novel hypothesis regarding mechanisms and implications for future vaccine development. Immunol. Cell Biol..

[128] Greinacher A., Selleng K., Palankar R., Wesche J., Handtke S., Wolff M., Aurich K., Lalk M., Methling K., Volker U. (2021). Insights in ChAdOx1 nCov-19 Vaccine-induced Immune Thrombotic Thrombocytopenia (VITT). Blood.

[129] Othman M., Labelle A., Mazzetti I., Elbatarny H.S., Lillicrap D. (2006). Adenovirus-induced thrombocytopenia: The role of von Willebrand factor and P-selectin in mediating accelerated platelet clearance. Blood.

[130] Roman G.C., Gracia F., Torres A., Palacios A., Gracia K., Harris D. (2021). Acute Transverse Myelitis (ATM):Clinical Review of 43 Patients With COVID-19-Associated ATM and 3 Post-Vaccination ATM Serious Adverse Events With the ChAdOx1 nCoV-19 Vaccine (AZD1222). Front. Immunol..

[131] Rinaldi V., Bellucci G., Romano A., Bozzao A., Salvetti M. (2021). ADEM after ChAdOx1 nCoV-19 vaccine: A case report. Mult. Scler..

[132] Nasuelli N.A., De Marchi F., Cecchin M., De Paoli I., Onorato S., Pettinaroli R., Savoini G., Godi L. (2021). A case of acute demyelinating polyradiculoneuropathy with bilateral facial palsy after ChAdOx1 nCoV-19 vaccine. Neurol. Sci. Off. J. Ital. Neurol. Soc. Ital. Soc. Clin. Neurophysiol..

[133] Shin M., Hyun C.Y., Choi Y.H., Choi J.Y., Lee K.H., Cho Y.S. (2021). COVID-19 Vaccination-Associated Lymphadenopathy on FDG PET/CT: Distinctive Features in Adenovirus-Vectored Vaccine. Clin. Nucl. Med..

